# Neurofilament Light Chain Correlates with Stroke Severity and Clinical Outcome in Acute Cerebrovascular Stroke Patients

**DOI:** 10.1007/s10571-025-01552-2

**Published:** 2025-04-30

**Authors:** Abeer Abdelhady Tony, Emad Farah Mohammad Kholef, Dalia B. Elgendy, Ahmed Shoyb

**Affiliations:** 1 Department of Neuropsychiatry, Faculty of Medicine, Aswan University, Aswan, Egypt; 2 Department of Clinical Pathology, Faculty of Medicine, Aswan University, Aswan, Egypt

**Keywords:** Acute ischemic stroke (AIS), Acute intracerebral hemorrhage (ICH), National Institute of Health stroke scale (NIHSS), Trial of ORG 10172 (TOAST) classification, Modified Rankin Scale (mRS)

## Abstract

Serum neurofilament light chain (sNfL) is a marker of injury in many chronic neurological Disorders. We assessed NF-L in patients with acute stroke (ischemic or hemorrhagic) and healthy controls and clarified its association with the stroke severity, etiology, and functional outcome. This case–control study was conducted on 85 patients with first-ever acute stroke (ischemic or hemorrhagic) and 85 control subjects. Participants were subjected to through neurological history and examination. Brain imaging was performed after hospital admission. Blood tests were drawn for assessment of serum neurofilament (sNfL) levels at the first day of admission. Compared to healthy controls, our stroke patients either Ischemic or hemorrhagic had increased sNfL levels. Despite not being statistically different, ischemic stroke patients had greater levels than hemorrhagic stroke patients. Higher sNfL levels were associated with higher NIHSS scores and mRS at admission. In patients with ICH, a correlation was observed between sNfL and hematoma volume, hemorrhage location, ventricular extension and mRS. Moreover, an association of sNfL with Ischemic stroke due to large artery atherosclerosis was reported. In the absence of additional predictive variables like age and sex, it may be possible to quantify sNfL in acute plasma samples as a potential indicator of functional prognosis in patients with ischemic and hemorrhagic strokes. To validate sNfL as a biomarker for acute stroke, however, further research involving a larger number of patients is necessary.

## Introduction

Stroke is a serious condition resulting in significant mortality and long-term morbidity. Axonal membrane breakage and neuronal injury resulting in brain damage cause the release of cytoskeleton proteins, including neurofilaments (NFs), into the interstitial fluid and ultimately into the blood and cerebrospinal fluid (CSF). NFs, or neuronal cytoskeletal proteins, are highly specialized structural proteins made up of alpha-internexin and four NF subunits: NF light (NF-L), NF medium (NFM), NF heavy (NF-H) chains (Nielsen et al. 2020). Serum NfL has been linked to a number of neurological conditions including frontotemporal dementia, motor neuron disease, and Alzheimer's disease (Duering et al. 2018). Serum neurofilament light chain (sNfL), a neuronal cytoplasmic protein vastly expressed in large myelinated axons, emerged as predictive biomarker in neuroinflammatory neurodegenerative disorders and showed promising results for monitoring neuroaxonal injury in patients with cerebrovascular disease (Rahmig et al. 2021). There have been attempts to choose appropriate prognostic indicators for post-stroke patients in an effort to better treat patients and allocate healthcare resources. Neuroaxonal injury is a major cause of long-term impairment following stroke and is critical to the functional prognosis and survival of stroke patients. Serum neurofilament light chain (sNfL) has recently been suggested as a marker of neuroaxonal injury after stroke with potential applications both for patient monitoring and for observational and interventional studies. The possibility of neurofilaments as hemorrhagic stroke biomarkers has not received as much attention as it has for ischemic stroke biomarkers. Only a few studies on patients with ICH are available (Cai et al. 2013), while the majority of the literature on hemorrhagic stroke examined neurofilament levels in patients with subarachnoid haemorrhage (SAH) (Lewis et al. 2008; Sellner et al. 2011). Although the neurofilaments were often assessed in the cerebrospinal fluid (CSF) and just a few days after the hemorrhagic stroke, the majority of these studies discovered a correlation between the levels of neurofilament heavy chain (NfH) and the clinical illness. Therefore, our goals were to: (1) ascertain the levels of sNfL in individuals who have experienced an ischemic or hemorrhagic cerebrovascular stroke; and (2) investigate the association between these levels and clinical stroke severity, functional outcome, hemorrhage size, location, and presence of ventricular extension.

## Patients and Methods

In this prospective, single-center case–control study, we enrolled patients presenting with first-ever acute stroke either ischemic or hemorrhagic and age-and sex-matched apparently healthy subjects as normal controls.

### Case Selection

The patients were enrolled from The Stroke Unit of the Neuropsychiatric Department, Aswan University Hospitals, between March 2022 and March 2023. Their ages varied from 20 to 88 years old, with a mean of 59.05 years. This will cover both genders. Patients with acute stroke (either ischemic or intracerebral hemorrhage) whose diagnosis was confirmed by computerized tomography (CT scan) and/or magnetic resonance imaging (MRI) of the brain. If the patients' symptoms appeared within a week and their ages > 18 years, they were eligible for the trial. Patients with brain neoplasm or other malignancies, history of previous stroke, other causes of intracranial hemorrhage (subdural, subarachnoid and extradural), hepatic/renal impairment, autoimmune diseases and vasculitis, endocrinal diseases other than diabetes mellitus, history of acute/chronic inflammatory diseases, history of trauma or surgery or acute vascular diseases that occurred within 4 weeks prior to the onset of stroke, were excluded.

### Study Tools

All patients were submitted for Complete medical and neurological history and examination; evaluation of Stroke etiology according to the criteria of Trial of Org 10172 in Acute Stroke Treatment (TOAST) classification and assessment of Stroke severity by using National Institutes of Health Stroke Scale (NIHSS) measured at the time of admission. Estimation of stroke Outcome by using the modified Rankin Scale (mRS), assessed at 30 days after stroke. Staging of hemorrhagic stroke was done according to ICH score (Hemphill et al. 2015). Brain imaging (either CT scan and/or MRI) was performed at admission where the volume, location of intracerebral hemorrhage and ventricular extension were assessed. The hemorrhagic volume was calculated by an expert radiologist through using the following formula: *A* × *B* × *C*/2 (*A* = greatest hemorrhage diameter in the axial plane, *B* = hemorrhage diameter at 90° to *A* in the axial plane, *C* = originally described as the number of CT slices with hemorrhage multiplied by the slice thickness, but can simply be substituted with the craniocaudal diameter of the hemorrhage where there is access to multiplanar reformats) (Kothari et al. 1996). Electrocardiography and echocardiography was conducted for all patients. 10 ml of venous blood has been collected from patients and control subjects and divided into: 2 ml in EDTA tube for complete blood picture; has been done on sysmex XP-300 cell counter and 8 ml divided on two plan tubes (for serum sample collection) for routine and special parameter estimation. Serum collected Samples have been left for clotting. All serum samples have been centrifuged at 3000 rpm for 20 min after clotting. Serum has been separated and divided into 3: One for routine parameter estimation (glucose, renal and liver function tests, calcium) all parameters has been done on fully automated chemistry analyzer BT-3500 (Italy); The other 2 divisions has been freezed at − 20 °CC for later use for estimation of Human Neurofilament Light Polypeptide (NEFL) level by ELISA Technique. All blood samples were collected on the first day of admission and haemolyzed samples has been excluded.

#### Principle of the NEFL Assay

Human Neurofilament Light Polypeptide (NEFL) estimation by Sandwich ELISA technique (GSCIENCE, Glory Science Co., LTD, USA). Kits adopted on coated microtiter plate with Human NEFL, make solid phase antibody, then add NEFL to wells (samples). Combine NEFL antibody with labeled HRP to form antibody-antigen-enzyme-antibody complex, after washing completely, add TMB substrate solution. TMB substrate becomes blue color at HRP enzyme-catalyzed, reaction is terminated by the addition of a stop solution and the color change is measured at a wavelength of 450 nm. The concentration of NEFL in the samples was then determined by comparing the optical density (OD) of the samples to the standard curve.

### Statistical Analysis

Data were analyzed using Statistical Program for Social Science (SPSS) version 24. Qualitative data were expressed as frequency and percentage. Quantitative data were expressed as mean ± SD for normally distributed data or median (IQR) for not normally distributed data. Mann Whitney *U* test (MW) was used when comparing between two groups (for abnormally distributed data) while for comparing between non-parametric data, Chi-square test was used. Kruskal Willis test (KW) was used when comparing between more than two groups (for abnormally distributed data). Pearson's correlation coefficient (r) test was used for correlating data. Receiver operating characteristic (ROC) curve was depicted to explore the diagnostic performance of s-NfL for diagnosis of stroke, analyzed as area under the curve (AUC), standard error (SE), and 95% CI. Validity statistics (sensitivity, specificity) were calculated. Test results were considered significant when *p*-value was ≤ 0.05.

### Ethical Aspect

The Institutional Ethics Committee of the Faculty of Medicine of Aswan University gave its approval to the current investigation (ASW.UNI.614/3/22). The goals of the research, the procedures, and the risk/benefit analysis were fully disclosed to each patient. When each subject was accepted to participate in the study, their signed permission was collected. According to the guidelines of the Declaration of Helsinki, the study was carried out.

## Results

Our study cohort consisted of 85 patients with acute stroke; 50 ischemic stroke patients, and 35 patents with hemorrhagic stroke. Male sex was the predominant sex (48, 56.5%), and median age was 54 years. One third of our stroke patients (28, 32.9%) were current smokers. Hypertension and dyslipidemia were the most predominant risk factors in our patients. Regarding stroke territory, there was a trend for a predominance of cerebral anterior strokes in our cases. Large artery atherosclerosis-related ischemic stroke was more common. In our patients with hemorrhagic stroke, supratentorial locus of bleeding was the most frequent site of bleeding. Our patients predominantly suffered from moderate cerebrovascular strokes assessed by NIHSS at admission. Based on their MRS at 30 days, they had a relatively poor prognosis (Table 1).

**Table 1:** Basic characteristics of studied cases

Parameters	Studied cases	
	Total no (85)	%
Age (years) (Mean ± SD, Range)	59.05 ± 15.4 (20–88)	
Sex (Male; No,%)	48	56.5%
Medical History (No; %)		
Smoking	28	32.9%
DM	32	37.6%
HTN	63	74.1%
AF	7	8.2%
IHD	2	2.4%
Dyslipidemia	39	45.9%
Clinical evaluation at admission		
Type of Stroke (No; %)		
Ischemic	50	58.8%
Hemorrhagic	35	41.2%
Circulation type (No; %)		
Anterior Circulation	77	90.6%
Posterior Circulation	8	9.4%
TOAST classification of stroke (No; %)		
Large artery atherosclerosis	25	50.0%
Small vessel occlusion	17	34.0%
Cardio-embolism	8	16.0%
Location of Hemorrhage (No; %)		
Supratentorial	1	2.9%
Infratentorial	34	97.1%
Hemorrhage volume (No; %)		
< 30 ml	20	57.1%
≥ 30 ml	15	42.9%
Ventricular extension (No; %)	9	25.7%
NIHSS (Range; Mean ± SD, median)	41, 13.4 ± 8.04 (12)	
ICH score (Mean ± SD, Range)	1.14 ± 1.2 (0–3)	
MRS (Range; median)	3 (1–5)	
sNFL (pg/ml) (Mean ± SD; Range)	433.1 ± 149.8 (203–1125)	

*DM* diabetes mellitus, *HTN* hypertension, *AF* non-valvular atrial fibrillation, *IHD* ischemic heart disease, *TOAST* Trial of Org 10172 in acute stroke treatment, *ICH Score* intracerebral hemorrhage (ICH) score, *NIHSS* The National Institutes of Health Stroke Scale, *MRS* Modified Ranking Scale, *sNFL* serum neurofilamint level

^*^Student *T*-test was used

**Fig. 1:**
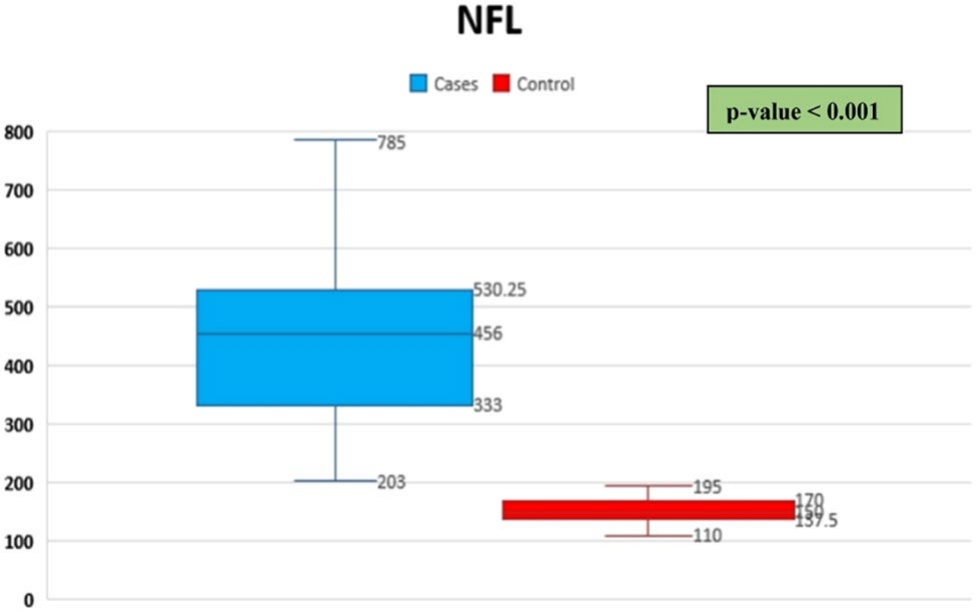
Comparison of sNFL between studied cases and controls

Table 2 shows highly statistically significant increased sNfL in studied Cases (median = 456, IQR = 333–530.25) when compared with Controls (median = 150, IQR = 137.5–170) with *p*-value < 0.001 (Fig. 1). Most of our cases (50 patients; 58.8%) had sNfL > 400 pg/ml. Individuals with ischemic stroke had statistically non-significant higher levels of sNfL than individuals with hemorrhagic stroke.

**Table 2:** Comparison of sNfL levels between studied cases and controls

Parameter	Control (*N* = 85)	Studied cases (*N* = 85)		*p*-value
NFL level Median (IQR)	150 137.5–170	456 333–530.25		< 0.001^*^
		Ischemic cases (No = 50)	Hemorrhagic cases (No = 35)	0.553
		470.75 327.25–542.5	453 336–527.5	

*Highly significant *p*-value. *p*-value < 0.001 is considered highly significant

MW: Mann Whitney *U* test that was used

In patients with hemorrhagic stroke, there was statistically significant correlation between increased sNFL and presence of intraventricular extension (median = 530.5, IQR = 466.7–621, *p*-value = 0.002) as well as hemorrhage volume ≥ 30 ml (median = 527.55, IQR = 466.5–563.5, *p*-value < 0.001). Also, it showed that there was high statistically significant correlation between sNFL and stroke severity assessed by NIHSS and stroke outcome assessed by MRS (*p*-value < 0.001, respectively). As well as. This table showed high statistically significant correlation between sNFL and large artery atherosclerosis as a cause for Ischemic stroke (median = 515, IQR = 491.5–606, *p*-value < 0.001) (Table 3).

**Table 3:** Correlation between sNfL level and clinical characteristics in studied cases

Parameters	sNFL Median (IQR)	*p*-value
NIHSS		
Mild (No = 13)	311 (262.7–354.2)	
Moderate (No = 66)	473.5 (340–529.2)	< 0.001^*^
Severe (No = 6)	618.2 (556.6–679.1)	
MRS		
No sig. disability (No = 8)	279.2 (255.3–345.1)	
Slight disability (No = 16)	318.2 (277.3–340)	< 0.001*
Moderate disability (No = 16)	358.7 (315–425)	
Moderate severe disability (No = 32)	503 (475.2–559.3)	
Severe disability (No = 13)	648 (529–679.7)	
TOAST classification		
Large artery atherosclerosis (No = 25)	515 (491.5–606)	
Small vessel occlusion (No = 17)	310 (271–339)	
Cardio-embolism (No = 8)	481.2 (384.1–639.7)	< 0.001*
Intraventricular extension		
No (No = 26)	372 (310.6–486.7)	0.002^*^
Yes (No = 9)	530.5 (466.7–621)	
Hemorrhage volume		
< 30 ml (No = 20)	341.5 (286.6–434.2)	< 0.001^*^
≥ 30 ml (No = 15)	527.5 (466.5–563.5)	

Mann Whitney *U* and Kruskal Willis tests were used

*NIHSS* The National Institutes of Health Stroke Scale, *MRS* Modified Ranking Scale

^*^p-value < 0.001 is considered highly significant

## Discussion

To the best of our knowledge, this is the first study compare the sNfL in Egyptian patients with ischemic and hemorrhagic stroke. In this instance, we provide the sNfL levels obtained throughout the acute and subacute phases (1st to 3rd days after the onset) after an ischemic and a hemorrhagic stroke in individuals for whom short-term outcome measurements (mRS) were accessible.

Compared to healthy controls, we observed that our stroke patients (Ischemic and hemorrhagic) had increased sNfL levels. sNfL levels increased in acute phase after ischemic insult because of the abundant neuroaxonal injury (Liu et al. 2020). Furthermore, our ischemic stroke patients were predictable to have these levels non significantly greater than hemorrhagic stroke patients. Our findings were in line with the results by Korley et al. who reported that baseline sNfL levels were higher in stroke subjects, compared to controls, after adjusting for age, race, blood pressure (Korley et al. 2019). Additionally, compared to healthy individuals, Gendron et al. revealed higher sNfL for all stroke types (acute cerebral infarction, ICH and aneurysmal SAH) (Gendron et al. 2020). Furthermore, our results were in agreement with De Marchis et al. who found that sNfL levels within 24 h of symptom onset were significantly higher in ischemic stroke compared to healthy controls (De Marchis et al. 2018). Moreover, Tiedt et al. found sNfL levels to be increased in ischemic stroke patients at admission (< 24 h), days 2, 3, and 7, and at 3 and 6 months' post-stroke, with peak levels at day 7 (Tiedt et al. 2018). Along with, Ahn et al. informed higher sNfL levels were in patients with acute ischemic stroke than those in age-and sex-matched healthy controls (Ahn et al. 2022). When comparing ICH patients to healthy controls, Zheng et al. found that there was a biphasic rise in sNfL, with a first peak occurring at roughly 24 h and a second spike occurring between days 7 and 14 after ICH (Zheng et al. 2023).

We found that sNfL levels are correlated with stroke severity assessed by NIHSS at admission and functional stroke outcome assessed by mRS at 30 days after stroke. Nevertheless, discrepant findings exist among studies evaluating early sNfL concentrations and functional outcomes of ischemic stroke. Whereas De Marchis et al. (2018) failed to detect associations of sNfL measured 24 h' post-stroke and 3-month mRS scores, Uphaus et al. reported that sNfL measured 24 h after admission did predict unfavorable 3 months mRS scores (Uphaus et al. 2019). Our results were consistent with previous studies explored the associations between sNfL at admission and stroke severity (Uphaus et al. 2019; Zhou et al. 2022; Chen et al. 2021). Moreover, Nielsen et al. reported that acute sNfL levels in ischemic stroke patients correlated with stroke severity assessed by SSS score on admission and with functional outcome (mRS score) 3 months' post-stroke (Nielsen et al. 2020). sNfL were measured in ischemic stroke patients within 30 days after the beginning of symptoms, Traenka et al. discovered that ischemic stroke patients had considerably higher sNfL levels, which were linked to a poor outcome at 3 months (Traenka et al. 2015). However, after controlling for NIHSS, this link became less significant, indicating that sNfL may be used as a prognostic biomarker during the acute phase. Conversely, Ahn et al. did not find a significant correlation between sNfL levels and stroke severity based on the NIHSS score on admission (Ahn et al. 2022). The limited sample size in this study may have contributed to the lack of statistical power and the observed difference. Furthermore, Moreover, Sellner et al. did not see a correlation between neurofilament levels and NIHSS, which might be explained by the small sample size and the use of neurofilament heavy chain (NfH) instead of sNfL (Sellner et al. 2011).

We discovered that the sNfL profile varies depending on the ischemic stroke etiologic subtype. The greatest acute phase concentrations were seen in LA strokes. Our results were consistent with the Pedersen et al. and Onatsu et al. researches (Pedersen et al. 2019; Onatsu et al. 2019). Arboix et al. (2010) described the clinical characteristics of patients with lacunar syndrome not due to lacunar infarct and identified clinical predictors of this variant of lacunar stroke. Their findings suggested that the pathophysiology, prognosis, and clinical features of lacunar strokes were different from those not due to lacunar infarcts. So, the function of sNfL in the ischemic lacunar stroke group in comparison to the non-lacunar ischemic stroke group should be investigated in future research.

Zheng et al. reported that The National Institutes of Health Stroke Scale, Glasgow Coma Scale, and hemorrhage volume all showed favorable correlations with sNfL levels in their patients with ICH (Zheng et al. 2023). Greater levels of sNfL within 72 h following ictus were shown to be independently linked to worsening functional outcomes (modified Rankin Scale ≥ 3) and higher rates of all-cause death at 6 and 12 months. Furthermore, they reported that sNfL levels within 72 h were closely associated with hematoma volume and disease severity, which was in line with our finding. Moreover, Cheng et al.; stated that sNfL was associated with baseline ICH volume and NIHSS (Cheng et al. 2020). Nevertheless, we discovered a substantial correlation between sNfL levels and the short-term outcome measured by mRS performed at 30 days, which contradicts their results that sNfL levels were predictive of long-term functional outcomes and death in patients with ICH. To the best of our knowledge, the location of the hemorrhage and ventricular extension were not examined in any of the prior researches on neurofilaments in hemorrhagic stroke. Apart from Zheng and his colleagues, who detailed positive correlation of sNfL with supratentorial location of hemorrhage, and cases of ICH without ventricular extension (Zheng et al. 2023). The severity of hemorrhage in patients with aneurysmal SAH (aSAH), and the volume of cerebral hemorrhage in patients with ICH were all connected with sNfL, according to Gendron et al. (2020).

Older age was significantly associated with sNfL levels in our stroke patients. We found no evidence linking sNfL levels to any other risk variables. In contrast to our findings, Korley et al. who established significant association of older age, nonwhite race, higher systolic BP, and higher hemoglobin A1C with sNfL levels (Korley et al. 2019). Moreover, he listed that sex was not associated with sNfL levels.

The study's strengths were as follows: first, patients were recruited concurrently from the same center and evaluated for two types of stroke as well as ACI subtypes using the same assay, allowing for direct group comparisons; second, a highly sensitive and well-validated assay was used to quantify sNfL (Kuhle et al. 2016).

The small number of patients included which decreased the power of some analyses and/or our ability to examine certain associations, the absence of longitudinal sampling (to evaluate the dynamics of plasma NF-L), and the lack of MRI diagnostics (patients had only CT and/or MRI during the acute phase) make up the limitations. Additionally, there is no information on a potential correlation between sNfL levels and infarct size. One other drawback is that there was no age adjustment applied to the correlations between sNfL and NIHSS or mRS.

## Conclusion

This explorative study concluded that sNfL was considered as a potential indicator of functional prognosis in patients with ischemic and hemorrhagic strokes. The higher sNfL levels were associated with higher NIHSS scores and mRS. Additionally, there was a link between sNfL and hematoma volume, hemorrhage location, and ventricular extension in ICH patients suggesting that sNfL levels assessed at hospital admission could be an indicative of hematoma volume and prompted more research on sNfL's potential as a biomarker in ICH. Likewise, it was discovered that sNF-L was linked to ischemic stroke caused by large artery atherosclerosis. However, more research including a greater number of patients and clot size measurement is required for this finding to be generalized. To validate sNfL as a biomarker for acute stroke, however, further research involving a larger number of patients is necessary. Additionally, research comparing sNfL levels across various intracerebral hemorrhage causes, such as traumatic cerebral hemorrhage, will be suggested. Furthermore, future studies should explore the role of serum neurofilament light chain in the ischemic lacunar stroke group compared to the non-lacunar ischemic stroke group.

## Data Availability

The corresponding author takes full responsibility for the data, has full access to all of the data, and has the right to publish any and all data separate and apart from any sponsor. This statement was mentioned in the methods section of the research.

## Abbreviations

NFs: Neurofilaments
CSF: Cerebrospinal fluid
sNfL: Serum neurofilament light chain
SAH: Subarachnoid haemorrhage
ICH: Intracerebral haemorrhage
TOAST: Trial of Org 10172 in acute stroke treatment
ICH Score: Intracerebral Hemorrhage Score
NIHSS: National Institutes of Health Stroke Scale
mRS: Modified Rankin Scale
CT: Computerized tomography
MRI: Magnetic resonance imaging
ELISA: Enzyme-linked Immunosorbent Assay
